# ‘I Live a Kind of Shadow Life’: Individual Experiences of COVID-19 Recovery and the Impact on Physical Activity Levels

**DOI:** 10.3390/ijerph182111417

**Published:** 2021-10-29

**Authors:** James Shelley, Joanne Hudson, Kelly A. Mackintosh, Zoe L. Saynor, Jamie Duckers, Keir E. Lewis, Gwyneth A. Davies, Ronan M. G. Berg, Melitta A. McNarry

**Affiliations:** 1 Applied Sports, Technology, Exercise and Medicine (A-STEM) Research Centre, Swansea University, Swansea SA1 8EN, UK; james.shelley@swansea.ac.uk (J.S.); joanne.hudson@swansea.ac.uk (J.H.); k.mackintosh@swansea.ac.uk (K.A.M.); 2 School of Sport Health and Exercise Science, University of Portsmouth, Portsmouth PO1 2UP, UK; zoe.saynor@port.ac.uk; 3 Department of Respiratory Medicine, University Hospital Llandough, Penarth CF64 2XX, UK; jamie.duckers@wales.nhs.uk; 4 Department of Respiratory Medicine, Prince Philip Hospital, Llanelli SA14 8QF, UK; k.e.lewis@swansea.ac.uk; 5 Faculty of Medicine, Health and Life Science, Swansea University Medical School, Swansea SA1 8PP, UK; gwyneth.davies@swansea.ac.uk; 6 Department of Biomedical Sciences, Faculty of Health and Medical Sciences, University of Copenhagen, Bledamsvej 3B, 2200 Copenhagen, Denmark; ronan@sund.ku.dk; 7 Department of Clinical Physiology and Nuclear Medicine, Copenhagen University Hospital, Rigshospitalet, Blegdamsvej 9, 2100 Copenhagen, Denmark; 8 Centre for Physical Activity Research, Copenhagen University Hospital, Rigshospitalet, Blegdamsvej 9, 2100 Copenhagen, Denmark

**Keywords:** exercise, SARS-CoV-2, long COVID, rehabilitation

## Abstract

Understanding of strategies to support individuals recovering from coronavirus disease 2019 (COVID-19) is limited. ‘Long COVID’ is a multisystem disease characterised by a range of respiratory, gastrointestinal, cardiovascular, neurological, and musculoskeletal symptoms extending beyond 12 weeks. The aim of this study was to explore individuals’ experiences of recovering from COVID-19 to provide a better understanding of the acute and long-term impact of the disease on physical activity (PA). Individualised semi-structured interviews were conducted with 48 adults recovering from COVID-19 at 6–11 months post-infection. An inductive thematic analysis approach was used, reaching saturation at 14 interviews (10 female; 47 ± 7 years). Four overarching themes were identified: (i) Living with COVID-19, including managing activities of daily living; (ii) Dealing with the Unknown and self-management strategies; (iii) Re-introducing physical activity; and (iv) Challenges of returning to work. The return to PA, whether through activities of daily living, work or exercise, is often associated with the exacerbation of symptoms, presenting a range of challenges for individuals recovering from COVID-19. Individually tailored support is therefore required to address the unique challenges posed by COVID-19.

## 1. Introduction

The novel coronavirus disease 2019 (COVID-19), resulting from infection with Severe Acute Respiratory Syndrome Coronavirus 2 (SARS-CoV-2), was first described in December 2019 [1]. In the UK, approximately 10% of individuals who have tested positive for SARS-CoV-2 report persistent symptoms beyond three weeks, although confirmation of viral infection has not always been widely available and is not a prerequisite for a COVID-19 diagnosis [2]. In some cases, persistent symptoms can occur in individuals who experience relatively ‘mild’ acute illness and it is not yet understood why some individuals develop persistent symptoms whilst others recover fully [2]. Acute COVID-19 is characterised by a range of symptoms, of which fever (98%), cough (76%), loss of smell and taste (62%), and myalgia or fatigue (44%) are most commonly reported, with less common symptoms including sputum production (28%), headache (8%), haemoptysis (5%), and diarrhoea (3%) [3,4]. Post-COVID-19 syndrome refers to signs and symptoms developed during, or after, an infection with COVID-19, which continue for more than 12 weeks [5]. The term ‘Long COVID’ is commonly used to describe both ongoing symptomatic COVID-19 and post-COVID-19 syndrome [5]. At six months after acute infection, 76% of individuals recovering from COVID-19 reported at least one persistent symptom, with fatigue being most commonly reported (63%) [6]. Individuals recovering from infection with severe acute respiratory syndrome coronavirus reported residual impairments in physical function one-to-two years post-infection and a similar prolonged response is anticipated for SARS-CoV-2 [7].

The range of neurological, musculoskeletal, cardiac, and respiratory symptoms experienced by individuals recovering from COVID-19 may impair their ability to be active, and an understanding of the role of exercise and physical activity (PA) in rehabilitation post-COVID-19 remains limited. Whilst many individuals recovering from COVID-19 report similar symptoms to those experienced by individuals with Myalgic Encephalomyelitis/Chronic Fatigue Syndrome (ME/CFC), it cannot be assumed that recommendations for graded-exercise therapy, which are appropriate for those with ME/CFC, will also be appropriate for individuals recovering from COVID-19 [8]. Indeed, the National Institute for Health Research (NIHR) recently highlighted a need for research to enhance understanding of the recovery from COVID-19 by listening to the lived experiences of sufferers, which focuses not only on exercise capacity but also PA [8]. The long-term impact of COVID-19 on PA is not yet fully understood, although a recent study found that self-reported walking is reduced up to six months post-infection, compared with pre-COVID-19 levels [9]. Additionally, participants reported engaging in less variety of activities, with 44% and 12% of participants reporting being unable to engage in any PA at three and six months post-infection, respectively [9]. Moreover, Delbressine et al. [9] reported that COVID-19 negatively impacts individuals’ ability to undertake activities of daily living (ADL) at three and nine months post-infection [9]. To date, only one study has explored the lived experience of individuals recovering from COVID-19 and any impact on their daily PA [10], highlighting the significant symptom burden and the challenges of managing COVID-19 during the post-acute COVID-19 period of recovery (three-weeks post-infection) [10]. There remains, however, a need to understand the longer-term impact of COVID-19 on individuals’ daily PA, including ADL, activity at work, leisure activity, and exercise. The aim of this study was to explore individuals’ experiences of recovering from COVID-19 to provide a better understanding of both the acute and long-term impact of the disease on their daily PA levels.

## 2. Materials and Methods

### 2.1. Participants

Overall, 48 adults recovering from COVID-19 (41 female; 47 ± 10 years; BMI 27 ± 7 kg·m^−2^) who were 6–11 months post-COVID-19 infection consented to participate and took part in semi-structured interviews. All were part of a larger sample (*n* = 281) participating in a randomised control trial investigating the use of inspiratory muscle training (Clinical trial number: 48075; Health and Care Research Wales Portfolio), in which they were eligible to participate if they were ≥18 years and had a previous infection with SARS-CoV-2, albeit a positive pathological test was not mandatory due to inconsistent testing availability in the UK at that time. Exclusion criteria comprised lacking capacity to understand the study protocol, or any pre-existing condition which would prevent them from exercising (e.g., cardiovascular disease).

### 2.2. Data Collection and Analysis

Ethics approval was granted by the Health Research Authority, Health and Care Research Wales (20/HR/3536), and local University Research Ethics Committee. Participants were recruited from the UK via social media and University webpages. Fully informed written consent and additional verbal consent were obtained prior to study commencement. Individualised semi-structured interviews were conducted to explore participants’ experiences of COVID-19, any perceived impact on their daily PA, and any adopted coping strategies. All interviews were conducted and recorded via online video conferencing software (Zoom Video Communications, San Jose, CA, USA) by a trained researcher and with participants in their own homes, and transcribed verbatim.

An inductive thematic analysis approach was used, whereby themes relating to participants’ experiences of PA post-COVID-19 infection were identified from codes applied to relevant quotations in the interview transcripts without using a predetermined framework. This approach was considered most appropriate given the paucity of existing research regarding this phenomenon. The analysis process used the six steps identified by Braun and Clarke: data familiarisation by repeated reading of transcripts whilst noting initial ideas for codes; identifying initial codes that relate to the research topic—physical and general activity; identifying themes to represent the coded data; reviewing themes to determine how well they fit together and reorganising codes within and between themes where appropriate; labelling themes to reflect their meaning and definition; and producing a written account of the themes that makes use of extracted quotes from the interviews to illustrate the integrity of the themes [11]. Tracy’s [12] ‘Big-tent’ criteria were used as markers of quality of the research process, illustrated within Table 1, with examples of how we used them to guide our research. Analyses were conducted until data saturation, the point at which additional analysis produced no additional codes or information [13], was deemed to be reached. The first 10 interview transcripts were manually coded, at which point it appeared that the saturation of codes identified was reached [14]. An additional four transcripts were then randomly selected, in a stratified manner, to ensure that interviews conducted at the start, middle and end of the interview process were included and there were no additional codes which could be identified. Moreover, this approach, importantly, identified that data saturation, whereby sufficient data have been analysed to understand the meaning of the codes [14], had been achieved. Codes were then discussed with a second researcher (KAM) and organised into themes.

## 3. Results

**Figure 1:**
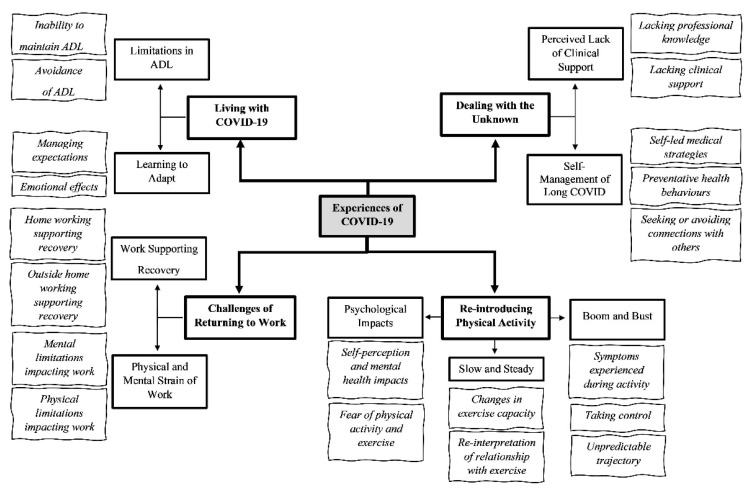
Displaying the four overarching themes (bold text and box), sub-themes (standard font and bold box), and codes (italic text) identified from semi-structured interviews exploring individuals’ experiences of COVID-19 and the perceived impact on physical activity.

Data saturation was deemed to be reached following the analysis of 14 of the 48 interviews (10 females and 4 males; 47 ± 7 years; BMI 29 ± 9 kg·m^−2^). Four overarching themes relating to PA, including ADL, work, leisure, and exercise, were identified following participants’ discussions of their experiences recovering from COVID-19: Living with COVID-19; Dealing with the Unknown; Re-introducing PA; and Challenges of Returning to Work (Figure 1). Illustrative quotes are provided for each theme, male and female participants are denoted by an ‘M’ and ‘F’, respectively, and details relating to the length of their recovery are also provided for context.

### 3.1. Pre-COVID-19 Physical Activity

All participants described themselves as active prior to contracting COVID-19, although this varied across a wide spectrum. Most participants’ descriptions of their PA levels aligned with recommended guidelines (moderate-to-vigorous physical activity three to five days per week):

*‘Prior to getting COVID, I would have put myself in a fit category’ (F01; 7 months post-infection)*

However, this varied from individuals engaging in ultra-endurance events, to individuals living with pre-existing chronic health conditions, which meant that undertaking ADL and work-related activity had to be managed to ensure that they did not over-exert themselves:

*‘I couldn’t live without it, yeah…I guess it’s the thing that gives me joy in life; it’s an inseparable part of me’ (F14; 10 months post-infection)*

*‘I have a medical condition anyway, which does cause some fatigue…I do think that having had the pre-existing health condition has helped me quite a bit because my ability to be patient and just try and accept things as they are and do what I can. I already had to do pacing, for example, whereas a lot of people have been having to get their heads around that for the first time ever, that have got ill with kind of long COVID symptoms’ (F09; 10 months post-infection)*

Regardless of their pre-COVID-19 PA levels and perceived fitness level, all participants described finding even ADL challenging. Some were still experiencing these difficulties at the time of interview (6–11 months post-infection).

### 3.2. ‘A Part of Our Everyday Lives’: Living with COVID-19

#### 3.2.1. Limitations in Activities of Daily Living

Overwhelmingly, participants discussed their inability to maintain the same ADL as they had pre-COVID-19. This ranged from simply not being able to sit up on the sofa during the initial stages post-infection, to only being able to carry out a reduced amount of housework, gardening or childcare.

*‘Your body, it shuts down, you lift your arms up, every, everything you do, to go to the toilet, to make a cup of tea, is an effort’ (M02; 6 months post-infection)*

Breathlessness and fatigue were reported as the primary limitation to carrying out ADL, although the severity of each varied between individuals:

*‘…if I’m trying to move or do any activities, the breathlessness that’s the… problem’ (F05; 10 months post-infection)*

*‘Yeah, you are very, very, very tired and, for me to go to the shop… is, too much, couldn’t do it’ (M02; 6 months post-infection)*

Regardless of the extent to which their ADL were affected, the effect persisted for months following their initial COVID-19 symptom(s):

*‘I haven’t made a cup of tea since March… I haven’t done anything, I haven’t made a meal, haven’t made a cup of tea, I tried, oh my gosh, I tried changing a pillowcase, oh my gosh, that’s hard, it’s so hard. No seriously, it’s really hard. I folded up four jumpers…I folded four and I was short of breath’ (F05; 10 months post-infection)*

Others avoided some ADL because of anxiety that even this low level of exertion would trigger symptoms:

*‘I’m worried about that in terms of doing any sort of strength work, I think that, if I go shopping and have heavy shopping, I, I worry if that’s like a, a kind of trigger…’ (F04; 7 months post-infection)*

Worry of being re-infected from mixing with others who might have COVID-19 stopped some participants from carrying out daily activities and living as they had done prior to being infected:

*‘So, I mean I’ve been super cautious and although everyone has said “don’t worry you’ve had it”, I think I’ve probably been over cautious. I mean, because of that, I’ve hardly done any driving because I’ve just not been out and about and I’ve not done the usual, the shops or anything like that. I don’t think I’ve done a single supermarket shop. So, in that respect, I’m not doing the usual kind of everyday activities anyway because I don’t particularly want to go out.’ (F08; 10 months post-infection)*

Whilst some participants felt they had been able to return to their pre-COVID ADL, the majority had been unable to, or unable to consistently, partly because of the unpredictability of their recovery:

*‘…yeah, I don’t know, it doesn’t, it doesn’t make sense this illness in terms of what the specific triggers are when I push it, whether that’s, you know, a physical thing… I know it’s physical because I know that, when I was pushing it early on, I would end up back in bed’ (F04; 7 months post-infection)*

This led to a need for a modified approach to ADL to allow people to manage day-to-day. Thus, at the time of interview (i.e., 6–11 months post-acute infection) most respondents were still experiencing challenges with completing ADL.

#### 3.2.2. Learning to Adapt

Several participants discussed the use of a pacing strategy to help them manage their ADL. For instance, planning to complete activities in only one part of the day, completing activities in smaller chunks, or trading off some activities for others:

*‘It’s like, if you have the energy, you just, you suddenly find yourself doing more, but then you sort of do too much, so the pacing thing really has been quite crucial to, you know, being able to do most things’ (M13; 11 months post-infection)*

As a result, some participants had normalised their changed and reduced capacity for carrying out the ADL which they had completed with ease prior to contracting COVID-19; nevertheless, this was a source of frustration:

*‘It’s amazing how much you normalise avoiding stuff that you can’t handle’ (F05; 10 months post-infection)*

*‘I was so frustrated I just wasn’t able to do stuff’’ (F05; 10 months post-infection)*

Many expressed concern that their current state of health would not improve and might even worsen, although others shared their optimism for a full recovery to pre-COVID-19 fitness levels:

*‘I think when you go for weeks and weeks and there’s no real difference you can put your finger on, in terms of improvement, you just think, “Well, is this it now? Is this just me now?”’ (F09; 10 months post-infection)*

*‘I fully expected, at some point, I’ll start to feel better and that this, you know, there may or may not be a long-term effect on the lungs but, if there is, then this is something I can deal with’ (F03; 7 months post-infection)*

However, given the novelty and uncertainty regarding COVID-19, participants discussed the difficulties they experienced in obtaining appropriate medical advice to understand how to manage their recovery.

### 3.3. ‘So Frightening’: Dealing with the Unknown

#### 3.3.1. Perceived Lack of Clinical Support

Participants discussed feeling like they were left to manage their recovery alone, without guidance or support from healthcare professionals:

*‘Whenever I speak to the [general practitioner] GP, the poor GPs have not been any help at all, and I think they’re quite open. They say “we don’t know what’s wrong” (F08; 10 months post-infection)*

This is not to say that medical professionals were unhelpful, with some participants reporting more positive experiences:

*‘I’m very lucky in that the GPs have been understanding, although not actually been able to provide any solutions, but they have been understanding and not dismissive’ (F10; 7 months post-infection)*

Others, however, had a less positive experience in finding professionals who would recognise their condition:

*‘I found it a struggle talking to my doctors, I felt like I wasn’t being believed, like I was just, you know, making stuff up or bigging things up’ (F14; 10 months post-infection)*

Irrespective, a common experience was being tested for multiple health parameters, and for these to fail to identify any definable causes of their current condition:

*‘…I then contacted my doctor because I thought that was an issue with my iron levels, who took the iron with thyroid issues, the thyroid was also fine. My doctor said that she can’t do anything else. I contacted another doctor, had, a full range of blood tests and they came back fine as well. I also had a chest X-ray, which was also okay and now, the latest doctor, after, after all the blood tests and chest X-ray, is sending me to a post-COVID clinic…’ (F14; 10 months post-infection)*

Without guidance and advice from the medical profession, participants attempted to find their own ways to manage their condition.

#### 3.3.2. ‘Like, Nobody’s Really Interested’: Self-Management of COVID-19

##### Self-Led Medical Strategies

Although one participant received treatment from an osteopath and acupuncture from a Chinese Medicine practitioner, without medical input, participants used their own treatments, including self-administered physical therapy to deal with fatigue, monitoring oxygen saturation, and seeking information from the internet and scientific literature. One participant discussed using:

*‘...facial exercises and massages for your lymph areas and everything and that’s been proven to work with chronic fatigue, so for the last week, I’ve been trying to do that, you know with hot and cold complexes, compresses on your spine’ (F05; 10 months post-infection)*

##### Preventative Health Behaviours

Many of the participants adopted preventative health behaviours to support their recovery, such as eating more healthily, sleeping and resting, weight loss, abstaining from alcohol, taking vitamins and supplements:

*‘I’m taking some vitamins and I’ve got a, a sort of over-the-counter antihistamines, which seems to help, ah actually… when the breathing was at its worst, I, there’s a, an over the counter nasal spray for, I think it’s a steroid something you take for hay fever, I don’t really get hay fever but I get a touch of it when the pollution is bad’ (M13; 11 months post-infection)*

Participants discussed actively managing their mindset and expectations to cope with COVID-19, meditating or remaining positive, and, as one participant discussed, focusing on:

*‘…what am I in control of and what am I not in control of’ (F01; 7 months post-infection)*

##### Avoiding or Connecting with Others

Many participants sought support from others, either in person or online, walking with friends, or engaging in online support groups:

*‘...that’s been really helpful in terms of, you know, a bit of support for other people that are going through the same thing, because at, at the beginning, the scary thing was, why’s this happening, why is this not written about, what, you know, people are going to think I’m putting this on…’ (F04; 7 months post-infection)*

Some people used avoidance as a coping strategy, avoiding those people who did not follow COVID-19 rules, or avoiding online fora where posts were perceived as ‘distressing’ or ‘self-pitying’:

*‘I’ve seen some comments by people about like, “Well, if I had to live with this fatigue for the rest of my life I’d kill myself”, and I think, “Well, I live with fatigue and I will do for the rest of my life, so thanks for that”’ (F01; 7 months post-infection)*

Family and friends were important sources of social support to exercise, and to help deal with the anxiety that participants still experienced about COVID-19:

*‘I’ve built up a little network of friends and I walk with friends so that it takes my mind off what I don’t, what I’m so scared about it becoming too, I don’t want to start internalising. So rather than going out on my own and thinking about stuff I go out with friends and then I can just talk and do the exercise without…and take my mind off the illness really.’ (F08; 10 months post-infection)*

### 3.4. ‘I Can’t Rest Anymore’: Re-Introducing Physical Activity

#### 3.4.1. Boom and Bust

Without exception, study participants were keen to return to their pre-COVID-19 PA levels as soon as possible and, in the absence of information to the contrary, they expected to be able to do so. Not unreasonably, participants attempted to resume some PA after the acute phase of their COVID-19 infection abated; however, for all participants, these initial attempts at activity were much more difficult than they anticipated, and all felt that they had unexpectedly over-exerted themselves. Participants discussed feeling breathless and struggling when they tried to walk, needing to sleep after initial attempts at PA:

*‘…the first time I went for a walk was probably only two miles and I ended up going to bed for two to three hours afterwards’ (F06; 10 months post-infection)*

This experience came as a shock as participants attempted what was, for them, activity well within their normal capacity, but for some, even stimulated a return of symptoms or an exacerbation of experienced symptoms, often described as a ‘relapse’ or ‘crash’:

*‘…and we walked one lunchtime and then I walked home from work, which was three miles and then the next day, I tried to walk home again and I got halfway home and thought I was going to fall over, like collapse’ (F04; 7 months post-infection)*

*‘I went for a swim and then I had heart palpitations for about twenty hours, it was really unnerving and uncomfortable, so I rang my GP, she sent me off for an ECG, which was fine and it’s been followed up since then and I have no long term or nothing that’s a big deal… I basically didn’t do anything again for two weeks after that and the heart palpitations went away but I’ve been much more gentle on myself with exercise since then’ (F03; 7 months post-infection)*

They quickly realised that their expectations of returning to pre-COVID-19 PA levels so soon were unrealistic and, for all participants, the recovery process was much lengthier than anticipated. Frustratingly, recovery was not the linear progression towards previously experienced levels of health and fitness that participants expected. They described recovery as a process of progression and regression or ‘boom and bust’, making a return to pre-COVID-19 PA levels challenging due to the uncertainty of how they would respond to PA on any given occasion:

*‘I’ve never had a sustained period of feeling well enough for a long enough period of time that’s made me think that I’m ready to do that and I’m too scared that I take a step back again’ (F04; 7 months post-infection)*

Advice regarding a return to PA and exercise was often conflicting and not specific or tailored for individuals recovering from COVID-19:

*‘They told me to walk then for fifteen minutes and well, I tell you what, I struggled…walking down’s not so bad but walking up is, the hill, is like oh no, no, no, no, no, but I am improving and to what I was back in, I say, in May’ (M02; 6 months post-infection)*

*‘…lung specialist…she suggested that I shouldn’t get back in the water yet because she’s treating her patients as though they have a post-viral pneumonia and she said she would never suggest that somebody with post-viral pneumonia would swim’ (F08; 10 months post-infection)*

Several participants had, however, made a conscious decision to return to exercise; they viewed this as a way of progressing their own recovery and did not consider prolonged rest as a long-term option:

*‘Thought I can’t rest anymore and sleep all day and night so started pushing myself to do shorter runs, yoga and home exercise’ (F14; 10 months post-infection)*

The exacerbation of symptoms caused by any form of PA meant that those returning to being active did so in a staged manner, at a level that was substantially below their pre-COVID-19 baseline fitness. They set themselves small goals to achieve as their experiences of overstretching themselves led to the onset of symptoms:

*‘On Sunday I got a bit cocky and I let it [heart rate] creep up to a ‘massive’ 130 [beats per minute] and I think that’s what hammered me, to be honest, so when I go back I’ll keep it low. I don’t have any breathing difficulties doing that but obviously I haven’t got close to threshold or been anaerobic for nine months now’ (M12; 10 months post-infection)*

At the time of joining the study, most participants had re-introduced some form of activity to their day, but this had not been the case for all. For instance, one participant with a chronic health condition was still limited to short walks in the garden with their dog. Another had attempted to return to activity, but these attempts had led to symptom exacerbation and frightening experiences of breathlessness whilst walking so they limited themselves to ADL and weekly yoga:

*‘…the last time I went around the block I was a little bit short of breath before I began, had been for a couple of days, a little bit short of breath. And it was awful, I didn’t think I was going to get home. I was in quite a state by the time I managed to walk home. Just walking like a zombie and barely able to breathe and trudging to get home really. Which has frightened me in doing activity like that’ (F10; 7 months post-infection)*

#### 3.4.2. Slow and Steady

Participants who were active prior to infection discussed that, whilst they still were able to complete slow and, in some cases, even prolonged, bouts of exercise by the time of interview, short, sharp bursts of higher-intensity activity were challenging:

*‘I’m alright with endurance things, so I can run slow and steady, that’s fine. And I do a little bit of speedwork now, but I can’t get as fast as I was pre COVID’ (F06; 10 months post-infection)*

Running, cycling, and walking uphill were also either difficult or impossible and some respondents changed their usual mode of exercise to be able to be active. All participants discussed a relatively diminished exercise capacity compared with their pre-COVID-19 level, and again, this ranged from an inability to keep up with training partners or complete strength-based exercise, to an inability to walk more than 50 steps at a time:

*‘…in terms of, you know, power, I mean I don’t have the strength back that I used to have, you know, physically lifting things, you know, I used to do a few weights and stuff, you know, a few times a week, just at home but I haven’t been able to do that, I can lift the weights now but it’s just, it’s still too painful’ (M13; 11 months post-infection)*

Breathlessness remained the most common limiting factor and the need for extended recovery and rest following activity was frequently mentioned:

*‘If I run upstairs really quickly by the time I’ve got to the top of the stairs I have to sit down and I have to recover.’ (F08; 10 months post-infection)*

Although, in a minority, some participants were able to view these changes in exercise tolerance positively, for instance, shifting focus from speed to endurance and managing respiration had helped to improve one participant’s swimming:

*‘bizarrely, my swimming has been great this year, I don’t know why, I think I’m a bit faster actually, that kind of helps but, but I think swimming is also a very measured breathing actually… you can’t sprint when you swim in open water because you can never get out of breath because that’s, ruins the whole style, so it’s a very measured breathing’ (F03; 7 months post-infection)*

Others were more appreciative of their exercise, for example, reframing their achievement:

*‘I sort of accept the fact that running is hard, it’s harder now, but, I guess, I’m, you know, I guess I get, I get better sense of achievement because despite feeling worse, I’m still doing it’ (F14; 10 months post-infection)*

#### 3.4.3. Psychological Impacts

Most interviewees discussed a negative impact of COVID-19 on psychological factors related to PA and exercise. Many discussed their fear of being physically active, which originated from a range of sources, including the fear of over-exertion causing a regression in health or long-term damage or simply fear of not knowing how they will feel when they are physically active. Some wanted to avoid potentially harming or burdening others if they experienced symptoms whilst exercising and needing help. Others were fearful of contracting COVID-19 again so avoided exercising in gyms where there is no fresh air or outdoors where they perceived a higher risk of contracting COVID-19:

*‘With the germs [in gyms] today, you’ve got to be very cautious’ (M02; 6 months post-infection)*

*‘I stopped cycling, even though everybody seemed to take it up, because I was struggling with my breath after I experienced some symptoms and I also believed in my own head that if I was breathing more heavily by doing exercise around the other people I might be taking it in—whether that’s right or wrong I don’t know. That’s what I thought at the time and nobody else could tell me otherwise’ (M07; 10 months post-infection)*

Others discussed the impact on their psychological state of being unable to exercise, including feeling depressed, suicidal, and feeling desperate to be active again:

*‘…tried walking, the same thing happened and they [the GP] said, what you need to do is go back to like a five-minute walk on the flat and try not to overdo it, so you can try and go every day because my mood was dipping, so severe, severe depression’ (F01; 7 months post-infection)*

Some participants experienced changes in their self-image or experience of exercise which was no longer the source of reinvigoration it once was:

*‘So, it’s almost like everything’s gone on a go slow. I feel that some days I wake up and I almost feel like I’ve kind of jumped forward 20 or 30 years and I feel like a little old lady instead of the active mum that I was six months ago’ (F08; 10 months post-infection)*

### 3.5. ‘I Can’t Really Work’: Challenges of Returning to Work

#### 3.5.1. Physical and Mental Strain of Work

All participants discussed being unfit to work at some point during their recovery, for varied periods of time and with varied impacts. For some, the impact was lessened as they had seasonal or sedentary occupations, but for others, whose work was very active, the impact was still evident at the time of interview:

*‘…My stock-in-trade is writing and using and testing outdoor equipment, so suddenly I can’t… if you can’t walk up a hill, you can’t test a rucksack, you can’t test boots, you can’t use this stuff and if you can’t use your brain to write stuff...effectively, it’s rendered me incapable of working’ (M12; 10 months post-infection)*

Those able to return to work experienced physical or mental challenges, or both, being unable to keep up physically with colleagues or taking regular naps throughout the day, regardless of working at a slower pace:

*‘Months afterwards extremely fatigued and falling asleep at the desk/taking power naps’ (F14; 10 months post-infection)*

*‘…with my team in work, I’ve got a lot of youngsters and, you know, where I’m normally, you, you know, in the front, come on, let’s go, not now…So it’s, I, I’m the one that’s trailing and it’s, and that’s not me, you know, alright you’ve got to allow for age, I know, but I’ve always been the first, let’s go for it, let’s go for it, you know and now, I’m, I’m saying, like let’s catch up all the time’ (M02; 6 months post-infection)*

Participants also discussed an inability to concentrate for prolonged periods of time, to focus on screens, or function at the same level or speed as they had done previously:

*‘…with the brain fog, I… really am quite forgetful, like really badly, so I have to write everything down, so rather than stress about it... for work, I write everything and for every meeting, I’m just making notes on everything, so that has helped quite a lot’ (F11; 9 months post-infection)*

#### 3.5.2. Work Supporting Recovery

Unlike prior to the COVID-19 pandemic, many respondents were now working from home, thus reducing the amount of necessary incidental work-related PA during travel to work and during the working day. Some participants felt that this supported their recovery as they would not be able to cope with these usual physical demands of work:

*‘…the total hours I’m doing are probably about two thirds of what I would usually be doing and it’s all from home, so I don’t have the driving that I would usually have when I go to work, I don’t have the walking round an office that I would usually have, I’m not even going up and down the stairs more than once day and once I’m downstairs I stay downstairs, I’ve got my work laptop in the living room and I’m just doing what I can, very reduced workload’ (F09; 10 months post-infection)*

In contrast, others who had returned to working outside the home felt that this was beneficial by offering a way to be physically active to support their recovery:

*‘I’ve started parking further away, to get a bit of exercise, so parking about a mile away from work, so I have a mile, two-mile walk, like a mile there and a mile back, just to try and gradually increase my fitness levels a bit’ (F04; 7 months post-infection)*

## 4. Discussion

Following recovery from the acute infection with SARS-CoV-2, many individuals have experienced a range of persistent symptoms of varied frequency and severity. Recovery is seldom linear and is, instead, characterised by recurrent exacerbations of new and existing symptoms, primarily shortness of breath and fatigue [15]. Participants in the present study reported that the burden of managing the ongoing effects of COVID-19 has a profound impact on all aspects of their life, including work, leisure, and ADL. These findings are congruent with a recent survey of individuals with long COVID, in which 48% of participants reported that COVID-19 had negatively impacted their ability to engage in exercise and 40% reported a negative impact on their work [16]. Returning to work was also reported as an important aim for recovery in the present study and it has previously been suggested that the onset of a chronic health condition is associated with a higher likelihood of discontinuing paid work [17].

Participants in the present study reported developing coping strategies to adjust to the limitations imposed by COVID-19, which is often done in the absence of clinical support that may have been beneficial in providing psychological support and practical recommendations to enhance recovery and avoid cycles of recovery and exacerbation. However, little was known at this point regarding recovery from COVID-19 and there were no specific recommendations for PA or exercise for either the general population or individuals recovering from COVID-19. In an attempt to regain physical function and cope with the requirements of daily living, some participants discussed a gradual increase in PA towards their pre-COVID-19 levels, whether through work, leisure, ADL, or exercise, which represented a route to recovery or was a necessity based on their personal circumstances. Whilst this was successful for some, in the presence of post-exertional symptom exacerbation, graded-exercise therapy is not recommended for individuals recovering from COVID-19, and PA requires careful consideration [18]. For some, PA was associated with exacerbations of symptoms, was detrimental to recovery, and could be associated with frustration and concerns regarding long-term health. An emphasis on PA as part of a person’s day-to-day life rather than structured exercise training may be more appropriate for individuals recovering from COVID-19 who are looking to return to, or increase, PA levels [8].

The return to their pre-COVID-19 PA levels, whether through undertaking ADL, returning to work, leisure, or more structured exercise, appears to be particularly challenging for individuals recovering from COVID-19 and the response can be variable. These findings suggest that conventional rehabilitation programmes, such as pulmonary rehabilitation, may not be appropriate or will need to be modified to address the unique challenges posed by COVID-19, in particular managing fatigue and post-exertional malaise. Given the varied manifestations of COVID-19, including, but not limited to, respiratory, neurological, gastrointestinal and cardiovascular symptoms, it is imperative that any future interventions are tailored to individual cases. Additional research is required to explore the efficacy of rehabilitation strategies in the population and will be essential to supporting individuals recovering from COVID-19.

## 5. Strengths and Limitations

The qualitative methods used in this study facilitated an in-depth exploration of individuals’ experiences of COVID-19 and the influence on PA. The findings may have important implications for future interventions seeking to support individuals recovering from COVID-19 returning to PA. It is pertinent to note that participation was voluntary, and recruitment was primarily conducted via online advertising and therefore there is potential for a self-selection bias towards individuals already motivated to be physically active and individuals more actively engaged in using social media. Sample bias may also be influenced by the fact that the majority of participants were female, hence further research with more male participants is needed to explore any potential sex differences. Furthermore, the majority of participants were infected during the early phases of the pandemic when testing was not widely available. A confirmed positive test was therefore not required to participate in the study. Although the majority of participants reported having either a positive test or diagnosis based on clinical symptoms, the research team were unable to verify this. However, the generalisability of the results is not based on conventional statistical probability but rather on the detailed exploration of multiple perspectives from a diverse sample of individuals recovering from COVID-19 of varied age and recovery duration [19].

## 6. Conclusions

The aim of this study was to explore individuals’ experiences of recovering from COVID-19 to provide a better understanding of both the acute and long-term impact of the disease on their daily PA levels. The return to PA, whether through activities of daily living, work or exercise, is challenging and often associated with exacerbations of symptoms. Individuals recovering from COVID-19 require individually tailored support to address the unique challenges posed by COVID-19, particularly managing fatigue and recovery expectations.

## Figures and Tables

**Table 1:** Tracey’s “Big Tent” Criteria.

Criteria	Examples of Use within Current Research
(1) Worthy topic	The research is unquestionably timely, relevant, and important.
(2) Rich rigor	The sample is purposive and information-rich, producing dense accounts of their experiences.
(3) Sincerity	We reflected together as a research team on our own biases and shaped our analysis to acknowledge these; for instance, KAM acted as a critical friend to JH who conducted the main analysis, whilst JS and MAM reviewed suggested themes and representative quotations. This was enabled by, for instance, a transparent audit trail of the analysis maintained by JH and detailed transcriptions of interviews.
(4) Credibility	We present thick description and verbatim quotations, coupled with our triangulation through multiple analysts engaging with the data to present a credible account of the participants’ experiences.
(5) Ethical	Procedurally, our research adheres to ethical codes and processes, and we are also mindful of relational ethics by ensuring we authentically represent experiences of activity during recovery from COVID-19.
(6, 7, 8) Resonance, Significant Contribution, Meaningful Coherence	To a degree, these are discerned by the reader, but we aim to influence resonance and transferability through the use of evocative quotations, to present an interpretation of the practical and theoretical relevance of our findings in our Conclusion, and we present a clear and detailed account of our purpose, methods, and interpretation of findings for readers to assess meaningful coherence.
COVID; coronavirus disease	

## Data Availability

Study data can be made available upon reasonable request.
